# NMDA Receptor-Mediated Signaling Pathways Enhance Radiation Resistance, Survival and Migration in Glioblastoma Cells—A Potential Target for Adjuvant Radiotherapy

**DOI:** 10.3390/cancers11040503

**Published:** 2019-04-09

**Authors:** Adriana Müller-Längle, Henrik Lutz, Stephanie Hehlgans, Franz Rödel, Kerstin Rau, Bodo Laube

**Affiliations:** 1 Neurophysiology and Neurosensory Systems, Technische Universität Darmstadt, Schnittspahnstrasse 3, 64287 Darmstadt, Germany; adriana1986@gmx.de (A.M.-L.); lutz@bio.tu-darmstadt.de (H.L.); kiki.rau@googlemail.com (K.R.); 2 Department of Radiotherapy and Oncology, Goethe-Universität Frankfurt, Theodor-Stern-Kai 7, 60590 Frankfurt am Main, Germany; stephanie.hehlgans@kgu.de (S.H.); Franz.Roedel@kgu.de (F.R.)

**Keywords:** ionotropic glutamate receptors, DNA repair, CREB inhibitor, NMDAR subunit GluN2B, radiotherapy, LN229, U-87MG, memantine, ifenprodil, sulfasalazine

## Abstract

Glioblastoma is one of the most aggressive malignant brain tumors, with a survival time less than 15 months and characterized by a high radioresistance and the property of infiltrating the brain. Recent data indicate that the malignancy of glioblastomas depends on glutamatergic signaling via ionotropic glutamate receptors. In this study we revealed functional expression of Ca^2+^-permeable NMDARs in three glioblastoma cell lines. Therefore, we investigated the impact of this receptor on cell survival, migration and DNA double-strand break (DSB) repair in the presence of both, glutamate and NMDAR antagonists, and after clinically relevant doses of ionizing radiation. Our results indicate that treatment with NMDAR antagonists slowed the growth and migration of glutamate-releasing LN229 cells, suggesting that activation of NMDARs facilitate tumor expansion. Furthermore, we found that DSB-repair upon radiation was more effective in the presence of glutamate. In contrast, antagonizing the NMDAR or the Ca^2+^-dependent transcription factor CREB impaired DSB-repair similarly and resulted in a radiosensitizing effect in LN229 and U-87MG cells, indicating a common link between NMDAR signaling and CREB activity in glioblastoma. Since the FDA-approved NMDAR antagonists memantine and ifenprodil showed differential radiosensitizing effects, these compounds may constitute novel optimizations for therapeutic interventions in glioblastoma.

## 1. Introduction

Glioblastoma (WHO grade IV) is one of the most common and aggressive malignant primary brain tumor in humans [1]. Despite recent incremental advances in surgical approaches, radiotherapy (RT, i.e., subsequent irradiation with single doses of 2 Gy daily, cumulatively max. 60 Gy), and chemotherapy (i.e., temozolomide), the median survival of patients with glioblastoma is only about 15 months [2,3]. Thus, the treatment of glioblastoma remains one of the most challenging task in clinical oncology. The so far limited success of the current multimodal therapy is likely due to the diffuse nature of the tumor and the prominent resistance to ionizing radiation (IR) with a high degree of recurrent invasive growth of the glioblastoma cells after the treatment [4,5,6,7]. In view of this and the outstanding role of RT for the prognosis of patients with glioblastoma, radiosensitizing substances addressing both, the high inherent migratory and invasive behavior and the intrinsic DNA double-strand break (DSB) repair capacity upon IR of glioblastoma cells may become increasingly important to improve therapy and patient survival. 

Accordingly, with regard to a putative improvement of the efficacy of adjuvant RT, effects of the tumor microenvironment on glioma proliferation, invasion and survival should be considered [8]. One striking feature of glioblastoma is that glioma cells release excitotoxic levels of glutamate (Glu) into the extracellular space at the tumor margin which is a 100-fold excess as compared to normal levels (>100 µM) [9,10]. Consequently, the prolonged high levels of Glu lead to neuronal cell death in the surrounding nervous tissue, which offers more space for tumor growth [10] and induce seizures within the peritumoral border in the patients [11,12,13]. This active Glu release by glioma cells is mainly mediated by the system x_c_^−^ cystine/glutamate exchanger [11,14]. Interestingly, pharmacological inhibition of this system by the FDA-approved drug sulfasalazine (SAS) was shown to slow tumor growth and to extend survival of tumor-bearing animals [15] and has therefore been considered for a treatment of both, gliomas and associated epilepsy [16]. In the brain, Glu is the major excitatory neurotransmitter by activating post-synaptic neuronal glutamate receptors upon pre-synaptic release and is crucially involved in normal brain function, including cognition, memory formation and learning [17]. Physiological Glu concentrations have also been shown to regulate proliferation, migration, and survival of neuronal progenitor cells and immature neurons during brain development [18]. 

Glu action is mediated by metabotropic glutamate receptors (mGluRs) and ionotropic glutamate receptors (iGluRs). The iGluRs are divided into three families: *N*-methyl-D-aspartate (NMDA), α-amino-3-hydroxy-5-methyl-4-isoxazolepropionic acid (AMPA) and kainate receptors [17]. It was originally considered that glutamatergic signaling is limited to neuronal cells in nervous systems. However, over the past decade, growing evidence occurred that iGluRs are also expressed in non-neuronal tissue [19], where they also play an important role in the proliferation, apoptosis, and migration of cells by interfering with their intracellular signaling pathways [20]. The ability of uncontrollable propagation and migration characterizes neoplastic cells as well; therefore, Glu has been suggested as a potential growth factor in tumor development [21]. Consistent with this notion, experimental data have implicated an important role of iGluRs in malignant tumor invasion and progression [22,23]. Furthermore, glioblastoma cells abundantly express AMPARs [22,23,24,25] promoting survival, invasion and migration and enhancing their malignant phenotype [10]. However, increased Glu concentrations can also mediate necrosis in malignant gliomas [26]. Thus, the multitude of paracrine and autocrine effects of Glu in glioma biology have supported the rationale for a pharmacological targeting of AMPARs in the treatment of glioblastoma [10]. Indeed, iGluR antagonists have been shown to increase the lifespan of human xenografts in mice [27]. In contrast, the role of Ca^2+^-permeable NMDARs in glioblastoma cells is enigmatic and their pathophysiological significance is largely unknown. For example, several publications state that NMDARs are non-functional in glioblastoma cells [25,28]. However, other reports indicate a functional role of NMDAR-signaling pathways in glioblastoma progression [23,29,30] and in a xenograft study [31]. So far five distinct subunits are known to generate Glu-gated NMDARs (GluN1 and GluN2A-D; overview in [17]). Four subunits assemble to a heteromeric complex [32], which in the case of the GluN1/GluN2-NMDAR subtypes are gated by simultaneous binding of Glu and glycine [33]. Especially the GluN2A and B subunits have been implicated in neuronal survival and death [34]. Under physiological conditions in the brain, NMDARs are crucial for brain development and neuronal plasticity involved in learning and memory formation [35]. Activation of NMDARs is translated to the nucleus of neurons by signaling-cascades leading to the phosphorylation of the cAMP-responsive element binding protein (CREB) and activation of genes promoting survival [34]. Interestingly, a putative role of NMDARs in tumor progression has been recently re-vitalized by several reviews highlighting the potency of targeting NMDARs in tumor treatment [29,36,37,38]. 

In the present work, we analyzed the potency of NMDAR-mediated signaling as a promising therapy to combat glioblastoma with a focus on two pathognomonic features of their biology, i.e., cell migration/invasion and, associated with that, the apparent intrinsic resistance of glioblastoma cells to RT. We found in the glioblastoma cell lines LN229 and U-87MG that (i) Glu improved DNA double-strand break (DSB) repair and that (ii) blocking NMDAR-mediated glutamatergic signaling resulted in a decreased cell migration, survival and a sensitization to IR. In addition we could show that Glu activates the CREB pathway and that DSB repair is inhibited by a CREB antagonist. Furthermore, NMDAR inhibitors showed a radiosensitizing effect in LN229 and U-87MG glioblastoma cells. Thus, particularly the FDA-approved drugs memantine and the GluN2B subunit-selective NMDAR antagonist ifenprodil seem to be promising as adjuvant therapy besides RT.

## 2. Results

### 2.1. Functional and Immunohistochemical Characterization of NMDARs in LN229 Glioblastoma Cells

**Figure 1:**
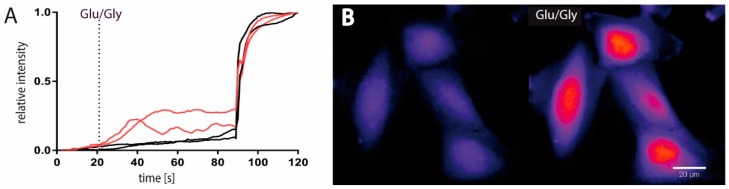

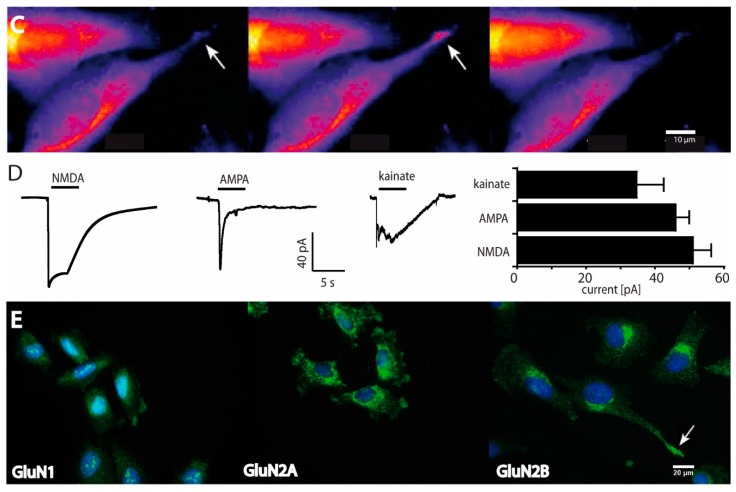
LN229 glioblastoma cells express functional NMDARs. (**A**) Ca^2+^-imaging of LN229 cells labeled with Fluo-4 AM displayed in ~50% of the cells a significant increase in relative fluorescence after glutamate/glycine (100 µM Glu/10 µM Gly) treatment (20 s). As an internal control, 10 µM ionomycin was given after 90 seconds. The integrated density of two non-responding (black) and two responding (red) cells are displayed. (**B**) Representative image of nuclear Ca^2+^-signals in four cells responding to Glu/Gly application (20 seconds before and after application). Scale bar 20 µm. (**C**) Images of Ca^2+^-signal oscillations in cell extensions (white arrows) 1, 4 and 10 seconds after Glu-application. Scale bar 10 µm. (**D**) Agonist-mediated traces and maximal inducible currents (*I*_max_) of kainate (100 µM), AMPA (100 µM) and NMDA (100 µM) analyzed by patch-clamp measurements (n = 22). Values of the *I*_max_ represent means ± SEM. (**E**) Immunostaining for GluN1, GluN2A and GluN2B NMDAR subunits with anti-GluN1, anti-GluN2A and anti-GluN2B (FITC, green) merged with DAPI (blue). GluN2B subunit expression in cell lamellipodia is indicated by white arrow. Scale bar 20 µm.

To analyze whether Ca^2+^-permeable ionotropic glutamate receptors (iGluRs) are functionally expressed in glioblastoma cells, we measured intracellular calcium concentrations ((Ca^2+^)_i_) in LN229 cells upon glutamate (Glu) application. In ~50% of the cells tested, a robust transient increase in the relative intracellular fluorescence intensity in their cell bodies was obtained after application of Glu (Figure 1A). Interestingly, a prominent (Ca^2+^)_i_ rise was consistently seen in the cell nucleus (Figure 1B) and in the extensions of the responsive cells (Figure 1C) indicating both, a Glu-mediated generation of nuclear Ca^2+^-transients and a spatial expression of Ca^2+^-permeable iGluRs at cell extensions. To test which iGluRs are functionally expressed, we performed patch-clamp recordings upon application of Glu and the iGluR-subtype specific agonists AMPA, NMDA and kainate. Consistent with our imaging results, ~40% of the cells tested showed a robust Glu-mediated inward current that recovered after washout (Figure 1D). Application of NMDA, AMPA or kainate revealed a more differential contribution of the Glu-mediated inward currents with the highest maximal inducible currents (*I*_max_) generated by NMDA and AMPA (Figure 1D). Since it has been postulated that NMDARs are not functionally expressed in glioblastoma cell lines (see [25,28,37]), we further tested three additional human glioblastoma cell lines (U-87MG, T98G, LN428) for their ability to respond to NMDA application. Two cell lines (U-87MG and T98G) revealed similar NMDA-inducible currents as obtained in LN229 cells, indicating that functional NMDARs are more commonly expressed in glioblastoma cells. To further verify the subunit composition of the expressed NMDARs and to determine their cellular localization, we labeled LN229 cells with antibodies targeting the GluN1, GluN2A and GluN2B NMDAR subunits. As shown by immunohistochemistry, membrane-associated localization of all three NMDAR subunits was evident (Figure 1E). However, the GluN2B subunit exhibited the most ubiquitous expression with an extensive punctuate pattern at long processes which is indicative of NMDAR clustering at the edge of lamellipodia (Figure 1E). Thus, our results demonstrate in three glioblastoma cell lines (LN229, U-87MG and T98G) functional expression of NMDARs. In addition, NMDARs in LN229 cells are composed of GluN1, GluN2A and GluN2B subunits with an enrichment of GluN2B-containing NMDARs at cellular extensions.

### 2.2. NMDAR-Activation is Crucial for LN229 Cell Viability, Migration and Survival

**Figure 2:**
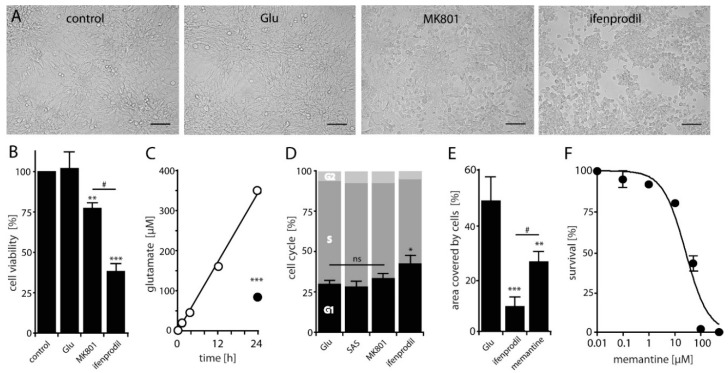
Effects of glutamate on LN229 cell viability, cell cycle distribution and migration. (**A**) Light microscope images of untreated cells (control), and in the presence of Glu (1 mM), MK801 (10 µM) and ifenprodil (25 µM). Scale bar 100 µm. (**B**) Cell viability measured after 48h by the MTT assay after Glu (1 mM), MK801 (10 µM) and ifenprodil (25 µM) treatment compared to control (untreated cells). The experiments were repeated at least five times performed in octuplet and values represent means ± SEM (one-way ANOVA followed by Bonferroni’s post-hoc test, ** *p* < 0.01, *** *p* < 0.001, ^#^ *p* < 0.05). (**C**) Increase in extracellular Glu concentrations of 3.5 × 10^5^ seeded cells at indicated time points (white circles) and after treatment with sulfasalazine (SAS, 250 µM, black circle) revealed a release of ~7.8 µg/mL Glu/h. Data are expressed as means ± SEM of three independent experiments performed in triplicate. Asterisks indicate a significant difference between treated and untreated cells as determined by Student’s *t*-test (*** *p* < 0.001). (**D**) Cell cycle distribution after 24h in the presence of Glu (1mM), MK801 (10 µM) or ifenprodil (25 µM) compared to SAS-treated cells (250 µM) (n = 4; one-way ANOVA followed by Bonferroni’s post-hoc test, * *p* < 0.05). (**E**) Cells were seeded for 48 h into two wells of an ibidi culture-insert for wound healing assays in the presence of ifenprodil (25 µM) and memantine (50 µM) compared to cells treated with Glu (1 mM). Data are expressed as means ± SEM of three independent experiments performed in triplicate. Asterisks indicate a significant difference between Glu-treated and antagonist-treated cells as determined by one-way ANOVA followed by Bonferroni’s post-hoc test, ** *p* < 0.01, *** *p* < 0.001, ^#^ *p* < 0.05). (**F**) Colony formation of cells treated with memantine revealed an LD_50_ value of 26 ± 11 µM. Data represent the means ± SEM (n = 3).

Our results so far indicate that functional NMDARs are expressed in three glioblastoma cell lines. Since it has been shown that iGluRs can induce Glu-mediated excitotoxicity in glioblastoma cells [26,39], we tested whether high concentrations of Glu may affect viability and survival of LN229 cells. We therefore incubated these cells with high concentrations of Glu and in the presence of the specific AMPAR antagonist Gyki-52466, the NMDAR antagonist MK801 and the GluN2B-subunit selective NMDAR antagonist ifenprodil and analyzed the morphology of the cells. Both, application of Glu and the AMPAR antagonist Gyki-52488 didn’t show any obvious morphological changes compared to the untreated cells whereas antagonizing NMDARs by MK801 or ifenprodil induced a reduction in cell number, a more rounded cell appearance and a reduced number of lamellipodia (Figure 2A). Notably, ifenprodil resulted in the most pronounced effect with a prominent retraction of cellular extensions (Figure 2A). Next, we measured cell viability by a colorimetric MTT assay in the absence and presence of the antagonists. Similar to the morphological effects, both, MK801 and ifenprodil significantly reduced cell viability compared to control with a significant higher effect seen with ifenprodil (Figure 2B) whereas cell viability was not affected upon Glu treatment. Based on these findings and the fact that glioblastoma cells can secrete high amounts of Glu [11], we speculated whether Glu-release by LN229 cells may cause constitutive active NMDARs in an autocrine manner. Therefore we analyzed the time-course of Glu-concentrations in the supernatant of LN229 cells (Figure 2C). The Glu-assay revealed that LN229 cells release Glu concentrations sufficient to saturate NMDARs already after 1h in culture which could be significantly blocked by the system x_c_^−^ cystine/glutamate transporter specific antagonist SAS (Figure 2C). Thus, our results indicate that i) LN229 cells release Glu in a high amount into the media and that ii) the changes in cell morphology and viability after blockage of NMDARs are likely due to a high concentration of endogenously released Glu. 

To test whether activation of NMDARs may influence the cell cycle progression of LN229 cells, we performed a cell cycle analysis after treatment with Glu and in the presence of SAS, MK801 or ifenprodil. Neither Glu nor diminishing Glu-release or blocking NMDARs by MK801 revealed differences in cell cycle distribution after 24h whereas treatment with ifenprodil resulted in a slightly increased cell population in G1 (Figure 2D) which unlikely contributes to the reduced cell viability seen in the MTT assay (see Figure 2B). However, since GluN2B-subunit containing NMDARs are expressed in lamellipodia (see Figure 1E) and MK801 slowed the growth of gliomas in situ [31], we wondered whether NMDAR antagonists influence cell migration. Therefore, LN229 cells were exposed to ifenprodil or memantine and the migration rate estimated for 48h. The antagonist-treated cells showed a significant stagnation of cell migration (Figure 2E), especially for the ifenprodil treated cells (Figure 2E). Based on this result we next examined the effect of memantine on cell survival by a clonogenic survival assay. Figure 2F shows a dose-dependent decrease in clonogenic survival for memantine normalized to untreated controls with an LD_50_ value of 26 ± 11 µM. A similar result was obtained with MK801 with an LD_50_ value of 0.9 ± 1.1 µM. Thus, our results revealed that treatment of the Glu-secreting LN229 cells with NMDAR antagonists can slow the growth and migration of cells, suggesting that activation of NMDARs in glioblastomas by ambient Glu may facilitate tumor expansion in vivo.

### 2.3. Antagonizing NMDARs Increases LN229 Radiosensitivity and Impairs Radiation-Induced DNA Double-Strand Break Repair

**Figure 3:**
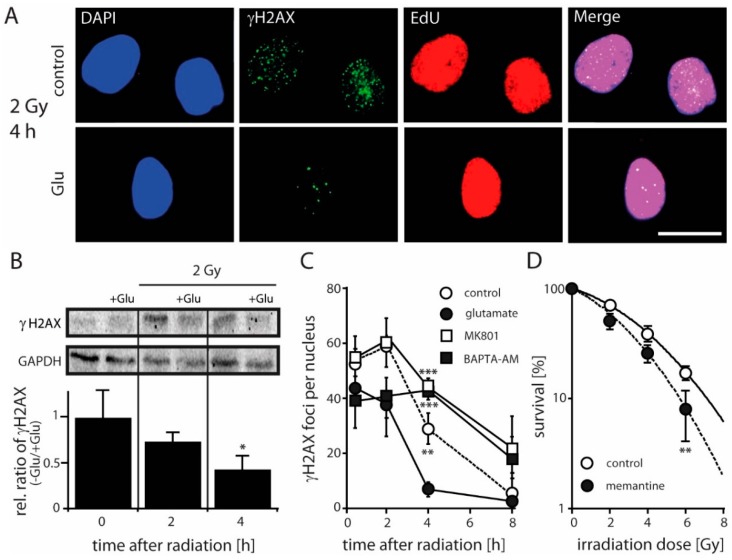
Effect of glutamate on DSB repair and clonogenic survival of LN229 cells. (**A**) Immunofluorescence of γH2AX, EdU and DAPI from Glu-treated and untreated cells 4 h after irradiation (2 Gy). Note that addition of Glu (1 mM) resulted in a significant decrease in DSB levels in EdU-positive cells compared to mock-irradiated control cells from 29 ± 5 to 7 ± 2 foci (*p* < 0.01; n = 3; Student’s *t*-test). Scale bar 20 µM. (**B**) γH2AX protein levels and their relative ratio (normalized to GAPDH expression) in the absence and presence of Glu (1 mM) before and after IR with a dose of 2 Gy detected by western blotting at the indicated time points (* *p* < 0.05; n = 3; Student’s t-test). (**C**) Repair kinetics of γH2AX foci in S/G2 phase cells after IR (2 Gy) under control conditions, in the presence of Glu (1 mM), MK801 (10 µM) and BAPTA-AM (3 µM). The difference between Glu-treated/untreated or MK801 and BAPTA-AM treated cells at the time point of 4 h is determined by one-way ANOVA followed by Bonferroni’s post-hoc test, ** *p* < 0.05, *** *p* < 0.001. (**D**) Colony formation of cells treated with memantine (50 µM) upon IR. The calculated radiation-induced cytotoxicity enhancement factor at 2 Gy is 1.4. Data are given as means ± SD of three independent experiments. The significant difference between treated and untreated cells is determined by Student’s *t*-test (** *p* < 0.01).

To evaluate the impact of NMDARs on the DNA repair capacity in glioblastoma cells, we used a well-established DSB-marker, the Ser139 phosphorylated histone H2AX (γH2AX) to stain for γH2AX in S/G2–phase LN229 cells. As shown in Figure 3A, adding Glu resulted in a pronounced decrease in γH2AX foci 4h after a 2 Gy exposure as compared to mock-irradiated control cells. To further elucidate the impact of Glu on DSB repair, we analyzed the relative levels of γH2AX in control and Glu treated cells upon IR by western blotting. Consistent with the decrease in the number of γH2AX foci, we found that Glu induced a significant decrease in the amount of γH2AX protein at 4h post IR (Figure 3B), suggesting that the presence of Glu results in a more effective DSB repair. In a next step we counted γH2AX foci in a kinetic covering 8h post IR in the presence of Glu and upon adding MK801. A pronounced increase of γH2AX foci was observed 0.5, 2, 4 and 8 h after 2 Gy radiation in MK801 treated cells, which was highly significant at 4h (Figure 3C). We next tested the impact of the Ca^2+^-chelator BAPTA-AM on γH2AX foci detection. Again, a highly significant increased number of γH2AX foci in BAPTA-AM-treated cells became evident at 4h after radiation (Figure 3C). Encouraged by these results and to test whether NMDAR antagonists are able to increase the sensitivity of LN229 cells to IR, we performed a clonogenic survival assay with cells treated with ifenprodil and memantine upon IR.

Both ifenprodil and memantine significantly reduced the 10% clonogenic survival rates in comparison to mock-treated controls (Figure 3D), resulting in a calculated radiation-induced cytotoxicity enhancement factor at 2 Gy of 1.81 and 1.4 for ifenprodil and memantine, respectively. Thus, our data indicate that antagonizing NMDARs by memantine or by the GluN2B-specific compound ifenprodil sensitize LN229 cells to radiation, likely by decreasing DSB repair capacity.

### 2.4. NMDARs Increase DSB Repair Capacity and Clonogenic Survival in LN229 and U-87MG Cells by Activation of the Transcription Factor CREB

**Figure 4:**
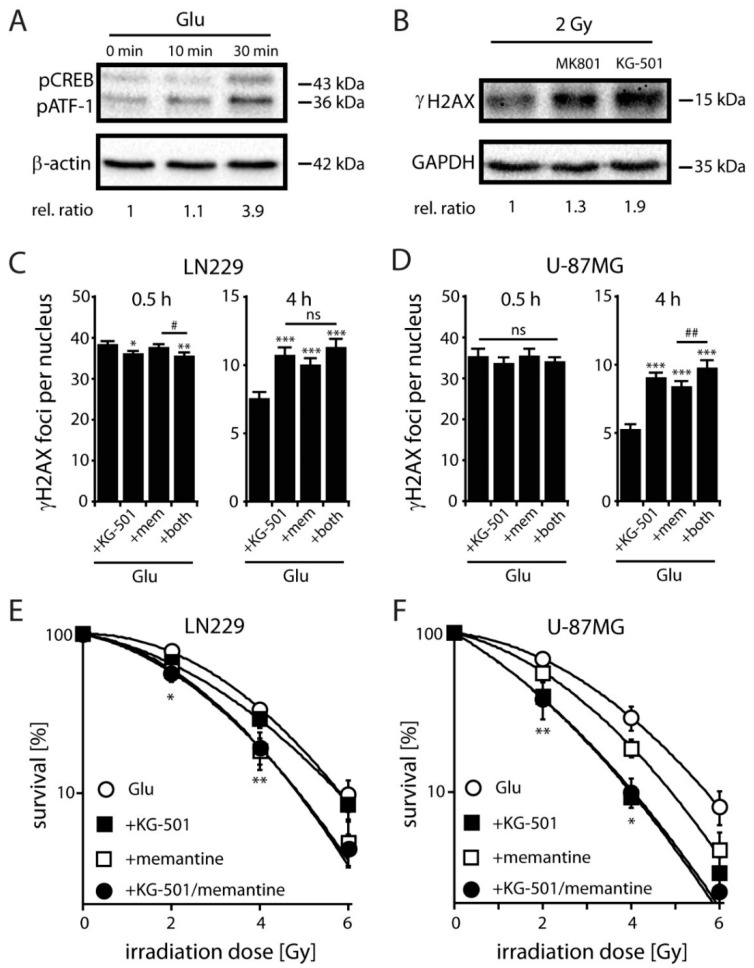
Effects of glutamate-induced pCREB on DNA repair and clonogenic survival in glioblastoma cells. (**A**) Relative pCREB levels in LN229 cells detected by western blotting (normalized to β-actin) at the indicated time points after treatment with Glu (1 mM). Note that the pCREB antibody is also detecting pATF-1, a protein closely related in structure and function to CREB (relative ratio at 30 min: 3.7 ± 1.1, *p* < 0.05; n = 3; Student’s t-test). (**B**) γH2AX protein levels and their relative ratio (normalized to GAPDH expression) in the presence of Glu (1 mM) and MK801 (10 µM) or KG-501 (25 µM) 4h after IR with a dose of 2 Gy detected by western blotting. Relative ratio for MK801 treatment is 1.4 ± 0.3 (ns; n = 3; Student’s t-test) and for KG-501 1.8 ± 0.4 (*p* < 0.05; n = 3; Student’s t-test). (**C**) γH2AX foci detection in EdU-negative non-S-phase LN229 cells at 0.5 h and 4 h following 2 Gy exposure after treatment with Glu (1 mM) and with Glu in the presence of KG-501 (25 µM), memantine (mem, 100 µM) and both inhibitors. Data represent means ± SEM of γH2AX foci of three independent experiments. Data were analyzed by one-way ANOVA followed by Bonferroni´s post-hoc test. * indicates significant difference between Glu-treated control and Glu in the presence of the antagonists, # indicates significant difference between the different antagonists (*^/#^ *p* < 0.05, ** *p* < 0.01, *** *p* < 0.001). (**D**) γH2AX foci detection in EdU-negative non-S-phase U-87MG cells at 0.5 h and 4 h following 2 Gy exposure after treatment with Glu (1 mM) and with Glu in the presence of KG-501 (25 µM), memantine (mem, 100 µM) and both inhibitors. Data represent means ± SEM of γH2AX foci of three independent experiments. Data analyses see Figure legend (**C**) (^##^ *p <* 0.01 *** *p* < 0.001). (**E**) Clonogenic survival of LN229 cells treated with 1 mM Glu in the absence and presence of KG-501 (1 µM), memantine (25 µM) and both inhibitors. Diagram shows fitted data. Memantine significantly reduces the survival starting at 2 Gy compared to Glu-treated cells (n = 3, each experiment was performed as triplet; error bars show SD; Student´s t-test, * *p* < 0.05, ** *p* < 0.01). (**F**) Clonogenic survival of U-87MG cells treated with 1 mM Glu in the absence and presence of KG-501 (1 µM), memantine (25 µM) and KG-501 and memantine. Both, memantine and KG-501 significantly reduce the survival starting at 2 Gy compared to Glu-treated cells. Note that co-application of KG-501 and memantine shows no significant further effect (n = 2, each experiment was performed as triplet; error bars show SD; student´s t-test, * *p* < 0.05, ** *p* < 0.01).

To understand mechanistically how NMDAR activation results in an altered DSB repair, we measured relative levels of the phosphorylated form of CREB at Ser133 (pCREB) in control and Glu-stimulated LN229 cells because CREB has been suggested to be involved in the resistance to IR [40]. We found that Glu induced a significant increase in the amount of pCREB protein after 30 min. (Figure 4A). To see whether activation of CREB through NMDARs can affect the expression of a downstream marker, we analyzed the induction of the brain derived neurotrophic factor (BDNF) gene in the absence of Glu and upon Glu treatment [34]. We found that Glu (1 mM) enhanced significantly the release of BDNF in LN229 cells (1.9 ± 0.15-fold, *p* < 0.05, n = 3, Student’s t-test) compared to the situation in the absence of Glu (SAS, 250 µM). The Glu-mediated increase in BDNF release could be efficiently blocked by MK801 (10 µM) and by the CREB specific inhibitor KG-501 [41] (25 µM). These data indicate that Glu can induce transcriptional-active CREB phosphorylation in LN229 cells via the activation of NMDARs. To further analyze the impact of NMDAR-mediated CREB activation on DSB repair, we analyzed the relative levels of γH2AX in MK801 and KG-501 treated cells upon IR in the presence of Glu (Figure 4B). We found that both antagonists induced a significant increase in the amount of γH2AX protein after 30 min irradiation (Figure 4B), suggesting that an effective DNA repair of radiation-induced DSBs in LN229 cells may depend on both, the activation of NMDARs and CREB. 

To further examine a possible involvement of CREB in Glu-induced radioresistance, we next analyzed DNA repair and clonogenic survival of LN229 and U-87MG cells in the presence of Glu and the CREB inhibitor KG-501 plus/minus memantine. We found an increased number of γH2AX foci after single KG-501 or memantine treatment in non-S-phase LN229 and U-87MG cells at 4 h after irradiation with 2 Gy, while 0.5 h after irradiation foci numbers were slightly decreased in LN229 and not changed in U-87MG cells upon inhibitor treatment (Figure 4C,D). Thus, our data revealed a significant increase of γH2AX foci in both, KG-501 and memantine treated cells after 4h compared to those treated with Glu alone. Double treatment with KG-501 and memantine only slightly increased the number of foci at 4 h after irradiation in comparison to single treatments, showing sub-additive effects of combined CREB and NMDAR inhibition (Figure 4C,D). Similarly, radiation survival of Glu-treated LN229 (Figure 4E) or U-87MG cells (Figure 4F) was only slightly further decreased upon combined treatment with KG-501 and memantine, highlighting a direct involvement of CREB in Glu-induced radiation resistance of glioblastoma cells and indicating that CREB is a key mediator in NMDAR-mediated DNA repair.

## 3. Discussion

Here, we present the first evidence that functional expression of Ca^2+^-permeable NMDARs in human glioblastoma cell lines is correlated with a radiation response. Our results show that glutamate improves upon activation of NMDARs DSB repair whereas inhibition by NMDAR antagonists impairs this process. Furthermore, we could identify a decisive role of NMDAR activity and subsequent downstream CREB activation in DSB repair since inhibition of CREB by KG-501 showed similar effects compared to NMDAR antagonists. Hence, our work demonstrates that NMDAR and CREB activation play an important role in IR-induced cellular damage in glioblastoma cells. In addition, treatment with the GluN2B subunit-specific NMDAR antagonist ifenprodil showed a decreased cell survival and cell migration resulting in a more pronounced radiosensitizing effect as compared to MK801, highlighting the clinical potential of GluN2B subunit-specific NMDAR-antagonists and CREB-mediated downstream signaling in optimizing RT in glioblastoma treatment. 

It has been shown that glioblastomas with a high Glu release have a distinct growth advantage [14,31]. Our results show that LN229 glioblastoma cells release glutamate into the media in an amount sufficient to activate NMDARs capable to induce NMDAR-mediated excitatory toxic effects [39,42]. Since we could not detect any Glu-induced excitotoxic effects in LN229 cells, we assume that this may reflect an increased capacity of LN229 cells to buffer intracellular Ca^2+^ allowing to survive prolonged Ca^2+^ influx. In contrast, we found a decrease in the viability of the LN229 cells when blocking glutamate release by SAS and by NMDAR antagonists, indicating that Glu induce NMDAR-mediated cell survival instead of cell death. Furthermore, when LN229 cells were cultured in the presence of SAS or NMDAR antagonists, the number of lamellipodia decreased, which might be suitable for tumor cell migration and invasion. In favor of our findings, suppression of Glu levels by SAS in orthotopic glioblastoma mice models prolonged survival and suppressed tumor growth in vitro and in vivo [15,24,43]. The potential of inhibiting the cystine/glutamate-mediated autocrine glutamate effect in glioblastoma treatment is also highlighted by a phase I clinical trial in newly diagnosed glioblasoma patients [10].

Concerning the target structures of an autocrine effect of Glu in glioblastoma biology, it has been shown that Glu stimulates the growth, migration and invasion of glioblastoma cells through activation of the AMPAR-subtype of iGluRs [22] and that iGluR antagonists increase the lifespan of human glioma xenografted in mice [27]. Other publications have shown a role of NMDAR-signaling pathways in tumor progression [23,29] and that NMDAR-antagonists have antitumoral effects when used in various xenograft tumors [44]. Here we show by Ca^2+^-imaging and patch-clamp recording that three glioblastoma cell lines (LN229, U-87MG and T98G) express functional NMDARs and that treatment of LN229 cells by blocking NMDAR-mediated glutamatergic signaling results in i) a decreased cell migration and survival and ii) a sensitization to IR. Thus, our results show that at least some glioblastoma cells express functional NMDARs and that stimulation with Glu elevates intracellular Ca^2+^ concentrations. Deregulated Ca^2+^ signaling is a prominent feature of pathological states, including those defined as “hallmark of cancer” [45]. Since NMDAR antagonists have been shown to inhibit migration of various types of tumor cells, including glioblastoma cells ([23,31]; this study), memantine constitutes a promising antagonist against “oncogenic NMDARs” [31], which is currently investigated in a phase I trial for post-radiation therapy in glioblastoma [46]. Interestingly, we further could show by colony formation assays and viability tests that blocking GluN2B-containing NMDARs by ifenprodil suppresses tumor cell survival more efficiently compared to the broad NMDAR antagonists MK801 and memantine. Based on these results we assume differential effects of NMDAR subunit-specific antagonists, indicating that subunit composition and/or membrane localization of the NMDAR subunits contribute different to glioblastoma malignancy. Indeed, we found a specific localization of the GluN2B-subunit in lamellipodia and a pronounced effect of the GluN2B-specific antagonist ifenprodil on migration of LN229 cells. Due to the high invasiveness of glioblastoma cells, our data are consistent with the idea of a specific role of GluN2B-NMDAR-signaling pathways at the invasive front of glioblastoma progression. This assumption is supported by the finding that grade 4 glioblastoma patients with high expression levels of GluN2B in the tumor have a worse prognosis [29] and underpin GluN2B-specific antagonists as an interesting therapeutic approach for treating brain tumors. 

IR-induced DSBs are the primary mechanism of tumor cell death [47]. Remarkably, we found a prominent role of the NMDAR and CREB in the modulation of the radiation response of LN229 and U-87MG cells. The effect of NMDARs and CREB on the repair of IR-induced DSBs is reflected by our finding that both, antagonizing NMDAR-mediated activation of CREB and CREB-itself increase the persistence of γH2AX foci after radiation in a non-additive manner. Thus, our results show that activation of NMDARs and the subsequent phosphorylation of CREB improve the repair of IR-induced DSBs and indicate that NMDAR-mediated downstream signaling via CREB plays a critical role in the protection of IR-induced cell damage in glioblastomas. In line with our findings, it has been shown that CREB is overexpressed in Acute Myeloid Leukemia (AML) cells and is associated with a poor prognosis [48]. The authors assume that CREB overexpression leads to chemotherapy resistance due to an increased DSB repair activity in AML cells. Expression of dominant negative CREB has also been demonstrated to reduce resistance to UV radiation in melanoma cells [49] and the tumorigenic potential in nude mice [50]. Remarkably, Amorino et al., 2003 described a direct link between CREB function and DSB repair [51]. Interestingly, the mechanism of radiosensitization included a reduction of the proliferating cell nuclear antigen (PCNA), a protein involved in the repair of IR-induced DNA damage [51]. However, CREB-activity increases also the release of BDNF in neoplastic cells) [34,52]. Interestingly, BDNF is thought to contribute to DNA damage repair (overview in [52]) and promotes survival and migration in C6 glioma cells [53]. Furthermore, Yano and colleagues (1998) have demonstrated in neuroblastoma cells that Ca^2+^-entry through NMDAR phosphorylates a serine/threonine kinase Akt, which activates CREB and results in the release of a variety of anti-apoptotic signals facilitating cell survival [54]. Thus, several data suggest an important role of CREB for cellular response to IR and the regulation of a presumptive transcriptional program that mediates tumor growth and invasion. However, how NMDAR-mediated activation of CREB affects tumor cell survival and the response to IR remains elusive. 

In conclusion, our work demonstrates that NMDARs and CREB play a role in the protection of IR-induced cell damage. CREB is phosphorylated in response to NMDAR-activation and the sensitivity of glioblastoma cells to IR is enhanced by the inhibition of both, NMDAR- and CREB-activity. Thus, further studies are needed to investigate NMDAR and CREB antagonism for enhancing tumor radiotherapy effects and to understand downstream effectors in glioblasoma physiology.

## 4. Materials and Methods

### 4.1. Cell Culture

Human glioblastoma cell lines LN229 (IDH1^wt^), U-87MG (IDH1^wt^; P53^wt^), T98G and LN428 were obtained from the American Type Culture Collection and were cultured in Dulbecco’s Modified Eagle’s Medium (LN229, DMEM, Sigma-Aldrich, Munich, Germany) or Minimum Essential Medium (U-87MG, T98G and LN428, MEM, Sigma-Aldrich) supplemented with 10% fetal bovine serum (FBS, Sigma–Aldrich), and 1% penicillin/streptomycin (Sigma-Aldrich). The cultures were kept at 37°C in a humidified atmosphere of 95% air and 5% CO_2_.

### 4.2. Chemical Treatment and X-Irradiation

LN229 and U-87MG were plated in cell culture dishes or well plates 24-48h before treatment. Chemicals were dissolved in ddH_2_O or DMSO (max. 0.2%). All antagonists (MK801, memantine, ifenprodil, BAPTA-AM (Tocris, Cologne, Germany) and KG-501 (Sigma-Aldrich) were applied in presence of the agonist Glu or NMDA (Sigma-Aldrich) and glycine (Roth, Karlsruhe, Germany) (if not stated otherwise) and maintained during the whole assay. All chemicals were added prior to irradiation. X-ray irradiation was performed at 90 kV and 19 mA with an aluminum filter with single doses of 2, 4 and 6 Gy using an x-ray tube equipped with a tungsten-anode (Philips, Amsterdam, Netherlands) as described previously [55]. X-ray treatment was performed using a power of 19 mA, 90 kV voltage and 30 cm distance to the IR source, which makes an applied dose of 1.96 Gy/min, established by Ficke-dosimetry. To all radiated samples equal control samples were performed and placed in the deactivated x-ray tube for the same time.

### 4.3. Measurement of Extracellular Glutamate and BDNF Concentrations

Extracellular Glu levels were detected using the Glu assay kit (KA1670, Abnova, Taipeh, Taiwan) according to the manufacturer’s protocol where glutamate is converted to 2-oxoglutarate via glutamate dehydrogenase reducing NAD^+^ to NADH. The subsequent oxidation of NADH drives a reaction catalysed by diaphorase, resulting in the conversion of *p*-INT to a formazan product, the absorption of which is measured spectrophotometrically. LN229 were grown to confluence in 60 mm dishes. Conditioned culture supernatant (250 µL) was collected after 2 h, 6 h and 24 h and analysed immediately. Collected media was transferred to a 96-well plate where glutamate dehydrogenase NAD^+^, and formazan were mixed. Absorbance of OD 565 nm was measured at time 0 and time 30 min using an Infinite M200 microplate reader (Tecan, Männedorf, Switzerland). The Glu exchange inhibitor sulfasalazine (SAS; 250 µM) was added to block release of Glu. The Glu concentration in the experimental medium was calculated via linear regression analysis (GraphPad Prism 7.0, GraphPad Software, San Diego, CA, USA) using defined Glu concentrations as standard. Measurement of extracellular BDNF concentrations: 2 × 10^4^ LN229 cells/well were seeded into 24 well plates and treated with sulfasalazine (SAS; 250 µM), 1 mM Glu, 20µM MK801 and 25 µM KG-501 directly after. The next day the supernatants of the cells were collected and centrifuged at 16200 g for 5 min. to remove remaining cells and debris. The BDNF concentration was determined with a BDNF ELISA Kit (ab99978, Abcam) following the manufacturer instructions. The absorbance at 450 nm was measured with the Tecan Infinite M 200 microplate reader.

### 4.4. 3-(4,5-Methylthiazol-2-yl)-2,5-Diphenyl-Tetrazolium Bromide (MTT) Assay

LN229 cells were plated at a density of 10^3^ cells in 96-well plates. 24 h after plating, cells were treated with memantine or MK801 and/or irradiation and after additional 48 h DMEM was supplemented with 10 µL of 3-(4,5-dimethylthiazol-2-yl)-2,5-diphenletrazolium bromide (MTT) reagent (5 mg/mL in PBS) to each well and incubated for 1 h at 37 °C. After discarding the MTT solution 150 µL isopropanol/0.04 N HCl was added and the plate was incubated till the formazan crystals were dissolved. The reaction product was quantified by measuring the absorbance at 570 nm with reference of 630 nm using the Tecan Infinite M 200 microplate reader.

### 4.5. Clonogenic Survival Assay

Clonogenic cell survival of LN229 and U-87MG cells treated with memantine, KG-501 or MK801 was analyzed by means of standard colony formation assay. Briefly, cells were trypsinized and plated at a constant cell density (500 cells per well in 6-well plate), and incubated for 7 days in the presence of memantine, KG-501 or MK801 with different concentrations. After 7 days of colony growth, cultures were fixed and stained with methylene-blue solution. The number of colonies formed with more than 50 cells were then determined and the plating efficiency (PE) and survival fractions (SF) were calculated. Calculation of SF was performed using the number of colonies of treated cells divided by that for the control cells seeded -(PE/100), taking into consideration the individual PE. Clonogenic cell survival of cells treated with memantine and KG-501 was analyzed after radiation (2, 4, and 6 Gy). The number of seeded cells was increased with irradiation dose (LN229: 400 cells/well form 0 Gy and 2 Gy, 800 cells/well for 4 Gy and 1600 cells/well at 6 Gy. U-87MG: 800 cells/well for 0 Gy and 2 Gy, 1600 cells/well for 4Gy and 3000 cells/well for 6 Gy). The cells were allowed to attach for 3 h and then irradiated in a X-ray tube as descripted above. Colonies were allowed to form for 8 (LN229) and 11 (U-87MG) days, fixated with 70% ethanol and stained with 0.1% crystal violet in 25% ethanol. The colonies with more than 50 cells were manually counted. The X-ray dose-survival curves were fitted by GraphPad Prism software version 4.0, to the linear quadratic equation, surviving fraction (SF) = exp(−αD − βD^2^), where D is the X-ray dose. The radiation sensitizing enhancement ratio (SER) by treatment was used to evaluate the drug-radiation interaction and calculated at a dose of 2 Gy using the following formula: SER = (SF2_control_)/(SF2_antagonist_). SER >1 suggests a radio-sensitizing effect. Each point on the survival curves represents the mean surviving fraction from at least two independent experiments performed in triplicate.

### 4.6. Western Blot Analysis

Samples were homogenized in 100 µL of whole-cell lysis buffer (150 M NaCl, 0.5% dodecylmaltosid, 50 mMTris, pH 7.5, 0.5% Triton X-100) mixed with complete protease inhibitor cocktail (Roche Diagnostics, Mannheim, Germany), and then lysed on ice. Equal amounts of protein samples (100 µg) were subjected to 14% SDS-PAGE and transferred to polyvinylidenedifluoride membrane (Amersham, Freiburg, Germany). Primary antibodies for γH2AX (Ser139) (JWB 301, Millipore, Schwalbach, Germany) and pCREB (Ser133) (Cell Signaling, Frankfurt, Germany) were used at 1:1000 dilutions. Secondary peroxidase- conjugated antibodies (Chemicon, Temecula, CA, USA and Santa Cruz, Heidelberg, Germany) were used at 1:10000, and the ECL Western Blotting Substrate (Pierce Thermo Fisher Scientific, Darmstadt, Germany) was used for visualizing the antibody-bound protein. To confirm equal protein loading, membranes were subsequently re-probed with anti-GABDH or anti- β-actin antibodies (Santa Cruz Biotechnology, Heidelberg, Germany).

### 4.7. Immunofluorescence Staining

LN229 cells (4 × 10^4^ cells per channel) were grown in µ-slides VI^0,4^ (Ibidi) over night, fixed with 4% PFA, permeabilized with 0.1% Triton X-100, blocked with 0.5% BSA/5% goat serum and incubated over night at 4 °C with following antibodies: anti-GluN1 (1:100, D65B7, Cell Signaling, Frankfurt, Germany), anti-GluN2A (1:100, N327A/38, Abcam), anti-GluN2B (1:200, S59-20, Stress Marq, Cadboro Bay, Victoria, Canada). After incubation with Alexa 488 labeled secondary antibodies (1:400, Abcam), the samples were stained with Hoechst 33342 and analyzed on an inverted epifluorescence microscope (Zeiss, Oberkochen, Germany).

### 4.8. γH2AX/EdU Double-Staining

LN229 and U-87MG cells grown on glass coverslips were treated with 10 µM EdU for 1 h to discriminate between S/G2- and G1-phase cells and irradiated with a dose of 2 Gy. Cells were fixed with 4% paraformaldehyde (PFA) for 10 minutes and permeabilized in 0.5% Triton X-100. Staining was performed with mouse-α-γH2AX antibody at 1:1000 (JWB 301, Millipore) and Click-it EdUAlexa Fluor 594 kit (Life Technologies, Darmstadt, Germany). Nuclei were counterstained with 4′,6-diamidino-2-phenylindole (DAPI) solution (Invitrogen, Karlsruhe, Germany) and coverslips were mounted with Mowiol (Roth). Images were taken using an AxioImager Z1 microscope and Axiovision 4.6. Micromanager software (Zeiss). For γH2AX foci quantification, 150–200 nuclei were evaluated for each data point from three independent experiments.

### 4.9. Fluorescence-Activated Cell Cycle Analysis

LN229 cells were grown in µ-slides VI^0,4^ (Ibidi) over night with/without glutamate/MK801 and treated with 10 µM EdU for 30 min. The cells were fixed with 4% PFA, permeabilized with 0.1% Triton X-100 and labeled with Alexa 594 azide using the Click-iT EdU Imaging Kit (Invitrogen). The samples were stained with Hoechst 33342 and imaged on an inverted epifluorescence microscope (Zeiss) and analyzed with micro manager. For this the integrated density of the Hoechst signal of single nuclei was plotted against the mean EdU signal and G1/S/G2 phases were gated manually. Mean ± SEM of at least three independent experiments were calculated and graphed. Analysis was performed on a FACScan (Becton Dickinson, Heidelberg, Germany) and data were analyzed using the ModFit LT 3.2 software (Verity Software House, Topsham, ME, USA).

### 4.10. Migration Assay

For measuring the change in the cell-covered area, over time as a parameter for migration rate we used culture inserts (Ibidi) consisting of two chambers. 2 × 10^4^ LN229 cells were seeded into each chamber in DMEM with 10% FBS. After 24 h the culture inserts were removed and quantification of migration was done by taking six pictures of the gap after 6, 24 and 48 h and calculating the area of the gap with ImageJ/FIJI software (http://fiji.sc/Fiji). Each experiment was done in triplicate.

### 4.11. Electrophysiology

Patch clamp recordings of ligand-gated whole cell currents were performed at −70 mV with the Port-a-Patch System (Nanion, Munich, Germany). Cells were trypsinized before recording and resuspended in external solution. Cells were sealed in solution containing (in mM) 80 NaCl, 3 KCl, 10 MgCl_2_, 35 CaCl_2_, 10 HEPES /NaOH, pH 7.4. For recordings a buffer with (in mM) 4 KCl, 140 NaCl, 2 CaCl_2_, 5 D-Glucose, 10 HEPES /NaOH, pH 7.4 was used as an external bath solution. The intracellular solution contained (in mM) 50 KCl, 10 NaCl, 60 K-Fluoride, 10 EGTA and 10 HEPES/KOH, pH 7.2. The sealing process and the access into the whole-cell mode were achieved with the help of PatchControl Software (Nanion). Solution exchange was executed by a rapid perfusion-system (Nanion). Currents were recorded with an EPC9 amplifier under the control of the Patchmaster Software (both from Heka Electronic, Lambrecht, Germany) as described previously [56]. Data were analyzed with Patchmaster and Fitmaster software (Heka Electronic).

### 4.12. Calcium Imaging

LN229 cells were seeded into 8-well µ-slides (Ibidi) and treated with the calcium dye Fluo-4 AM (Thermo Fisher) dissolved in DMSO loaded for 30 min. to a final concentration of 2 μM. The cells were washed once with the imaging-buffer (140 mM NaCl; 2.8 mM KCl; 1.8 mM CaCl_2_; 10 mM HEPES; 20 mM Glucose; 10 µM EDTA; pH 7.2) and imaged in a recording chamber on a epifluorescence microscope (Zeiss) at a rate of 1 Hz for 120 seconds. Alterations in fluorescence as a function of time were measured at a single wavelength. Glutamate and glycine were applied to the cells to an end-concentration of 1 mM and 100 µM respectively after 20 seconds and 10 µM ionomycin was given after 90 seconds. The image sequences were analyzed for visual inspection and processed by using *ImageJ* /FIJI software (http://fiji.sc/Fiji) by subtracting the mean background of the integrated density of every cell and then normalize all values to the fist and the highest value of every cell. The data were plotted as relative fluorescence intensity scale versus time.

### 4.13. Data Analysis

Experimental data are presented as mean ± SEM from three or more independent experiments (if not indicated otherwise). Levels of significance were calculated using the Student´s unpaired t-test or one-way analyses of variance (ANOVA followed by Bonferroni´s post-hoc test) (GraphPadPrism).

## 5. Conclusions

In conclusion, our findings in LN229 and U-87MG cells support a new approach for the therapy of brain tumors based on antagonizing NMDAR-mediated signaling pathways upon radiation resulting in (i) an enhanced radiosensitivity by suppressing DSB repair capacity and (ii) a decreasing tumor cell survival and migration.
